# Supplementary material for: Interactomic Analyses and a Reverse Engineering Study Identify Specific Functional Activities of One-to-One Interactions of the S1 Subunit of the SARS-CoV-2 Spike Protein with the Human Proteome
Source: Biomolecules. 2024 Dec 3;14(12):1549. doi: 10.3390/biom14121549 (PMC12121346; doi:10.3390/biom14121549)
Supplement: Supplementary file 1 [file biomolecules-14-01549-s001.zip › biomolecules-3267845-supplementary.pdf]

SUPPLEMENTS
to the article:

Interactomic Analyses and a Reverse Engineering Study Identify Specific Functional Activities of One-to-One Interactions of the S1 Subunit of the SARS-CoV-2 Spike Protein with the Human Proteome

Giovanni Colonna

Unit of Medical Informatics—AOU Luigi Vanvitelli, University of Campania, 80138 Naples, Italy; giovanni.colonna@unicampania.it

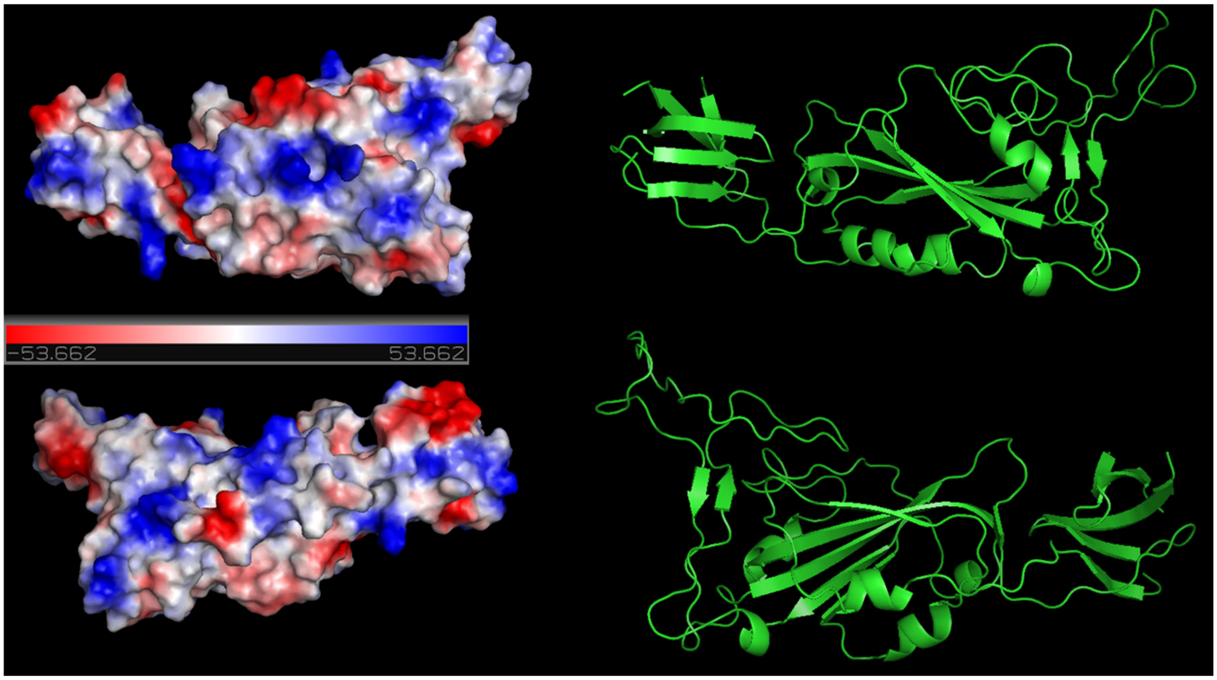

Figure S1. The figure shows the three-dimensional structure of the S1 fragment 320-590 from multiple views. The cartoon views (on the right) show two opposing perspectives of the structure. The disordered and flexible parts without a definable structure are clearly visible. On the left are the views of the charged surfaces (blue is positive) with the same perspectives. The protein shows an extended shape and predominantly positive surface charges at neutral pH. The long flexible segments contain epitopes. We generated the Poisson-Boltzmann charge surface for the S1 subunit by PyMOL. The structure of the fragment has been isolated from the PDB data of the ACE2-S1(fragment) dimer [240]. A molecular model showed that the S1 fragment of SARS-CoV-1 binds to ACE2 with a higher affinity than the full-length protomer.

Table S1. 158 LT proteins extracted from BioGRID

ACE2, AGTR1, APOA4, APOE, ARPC3, ASGR1, AVP, AVPR1B, AXL, BSG, BTN3A2, C3, CACNA1C, CALCOCO2, CAMP, CANX, CD207, CD209, CD33, CD44, CDK2, CDK4, CFB, CFH, CFI, CFP, CKAP4, CLEC10A, CLEC4G, CLEC4M, CLPTM1, CNTN1, COP1, COPB1, COPB2, CRP, CSNK1G3, CSPG4, CTSB, CTSK, CTSL, CTSS, CTSV, CYC1, CYCS, DPP4, EGFR, EPHA7, ESR1, EZR, F10, F2, FGB, FGG, FLT1, FSCN1, FURIN, GOLGA7, GOLM1, H3C12, HAVCR1, HBA1, HBA2, HBB, HLA-A, HP, HSPA5, HSPG2, IFITM1, IFITM2, IFITM3, IL12RB1, IL1RAPL2, IRF3, IRF9, ITGA5, ITGAV, ITGB1, ITGB3, ITGB5, ITGB6, KREMEN1, LAMC2, LDLR, LDLRAD3, LGALS3, LGALS7, LGALS8, LRRC15, LTF, LYPLA2, LYSMD3, MAL2, MARCHF8, MBL2, MPZL1, MRC1, MSMP, MSN, MYH9, NAPA, NCL, NEDD4, NGF, NID1, NRP1, NRP2, OCLN, PARP1, PIM2, PPIA, RAB1A, RAB1B, RAC1, RRAD, SCAMP3, SDC1, SDC2, SDC3, SDC4, SEC23A, SEC24C, SFTPD, SIGLEC1, SIGLEC10, SIGLEC9, SLC1A5, SLC6A15, SLC7A4, SMURF1, SMURF2, SNCA, SNX27, SNX5, SNX6, SPCS2, STAT1, TAT2, TFRC, TG, TLR4, TMEM106B, TMEM30A, TMPRSS2,TSSK3, TTYH2, TUBB8, VIM, VPS26A, PS35, WDR45B, WWP1, WWP2, ZC3HAV1, ZDHHC2, ZDHHC20, ZDHHC3, ZDHHC5.

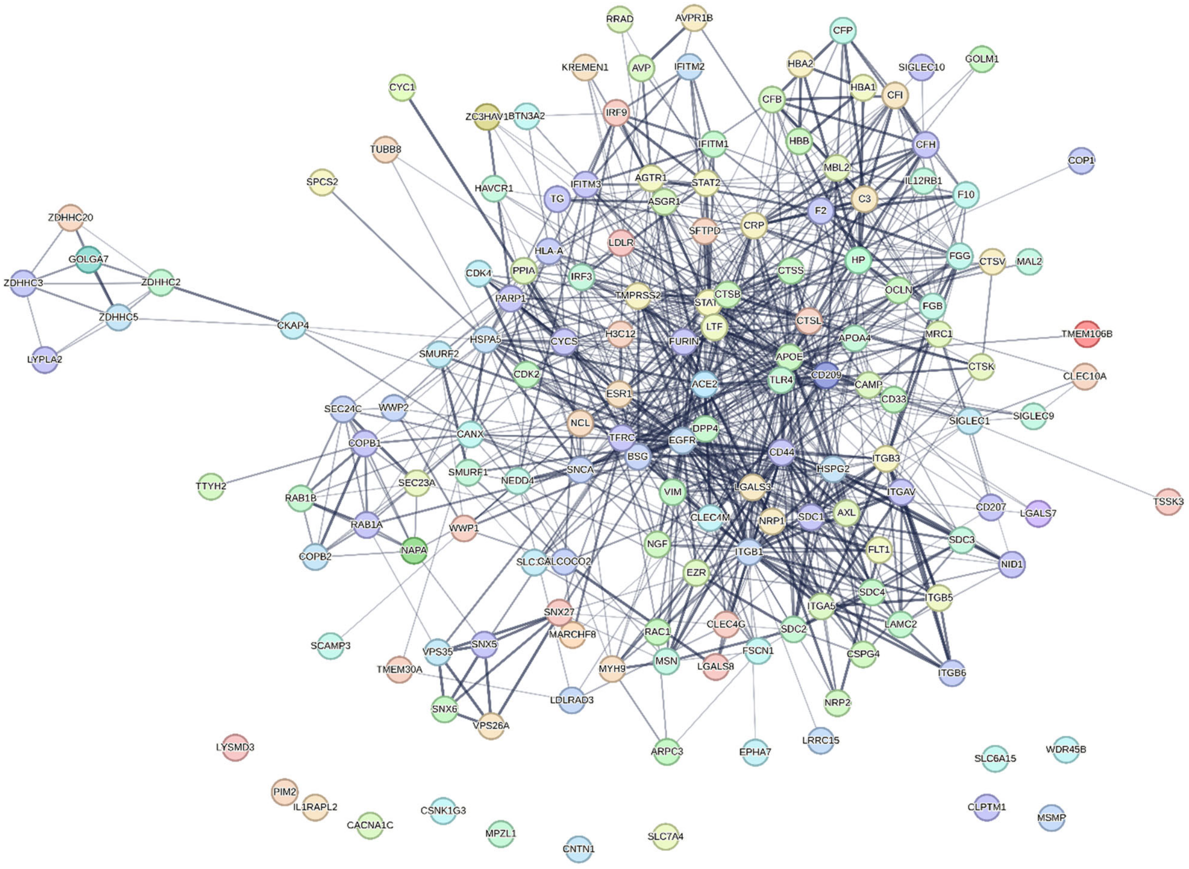

Figure S2. Interactome calculated by STRING for the 158 interactors of S1, selected by BioGRID. Most proteins appear to form a compact, dense network spontaneously, which suggests common biological activity. Confidence score: 0.400; all 7 channels open as an active interaction source; number of unconnected nodes: 12; *p*-value: < $1.0x10^{-16}$.

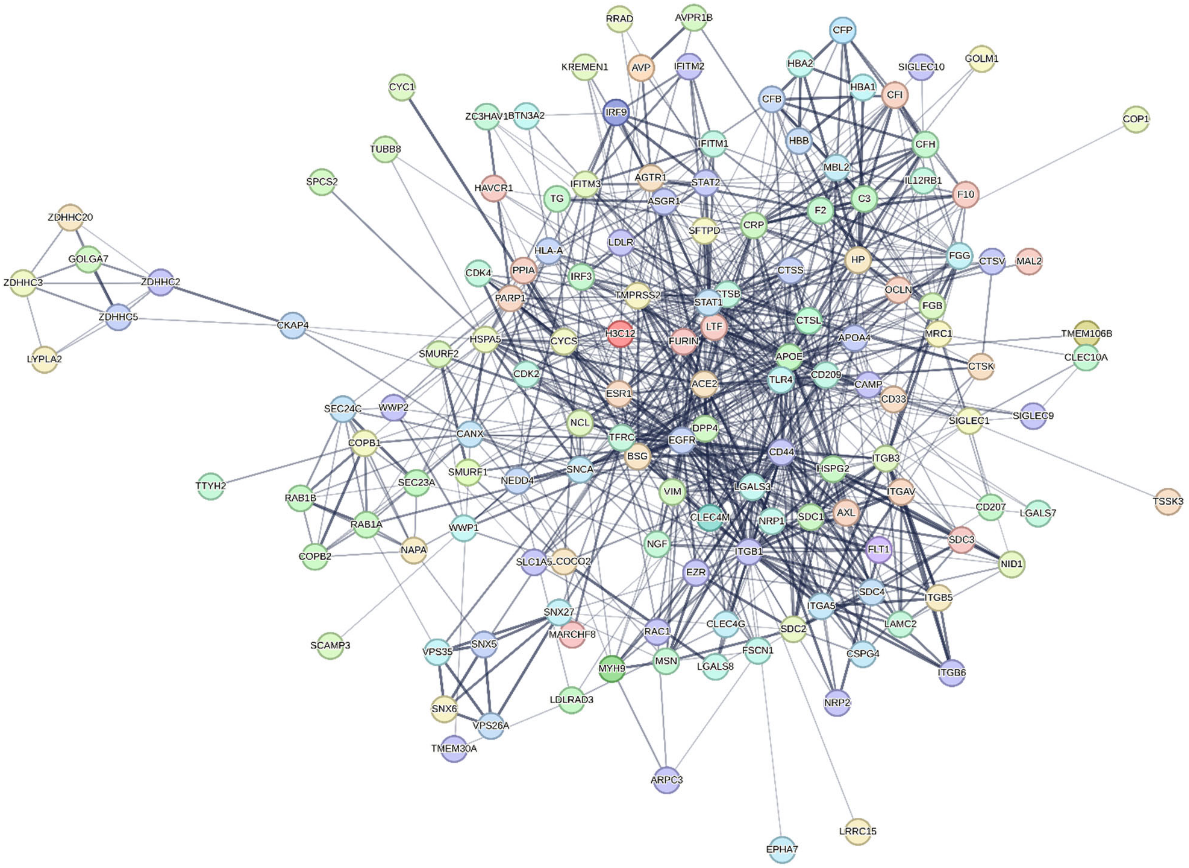

Figure S3. Pruned interactome of figure 1S. Confidence score: 0.400. Source channels: 7. Topological parameters - number of nodes: 146; number of edges: 901; average node degree: 12.3; avg. local clustering coefficient: 0.519; expected number of edges: 331; PPI enrichment *p*-value: < $1.0x10^{-16}$. Top five high-ranking nodes with degrees: EGFR (59), APOE (48), CD44 (47), TLR4 (45) and ACE2 (37). Topological parameters calculated by Cytoscape.

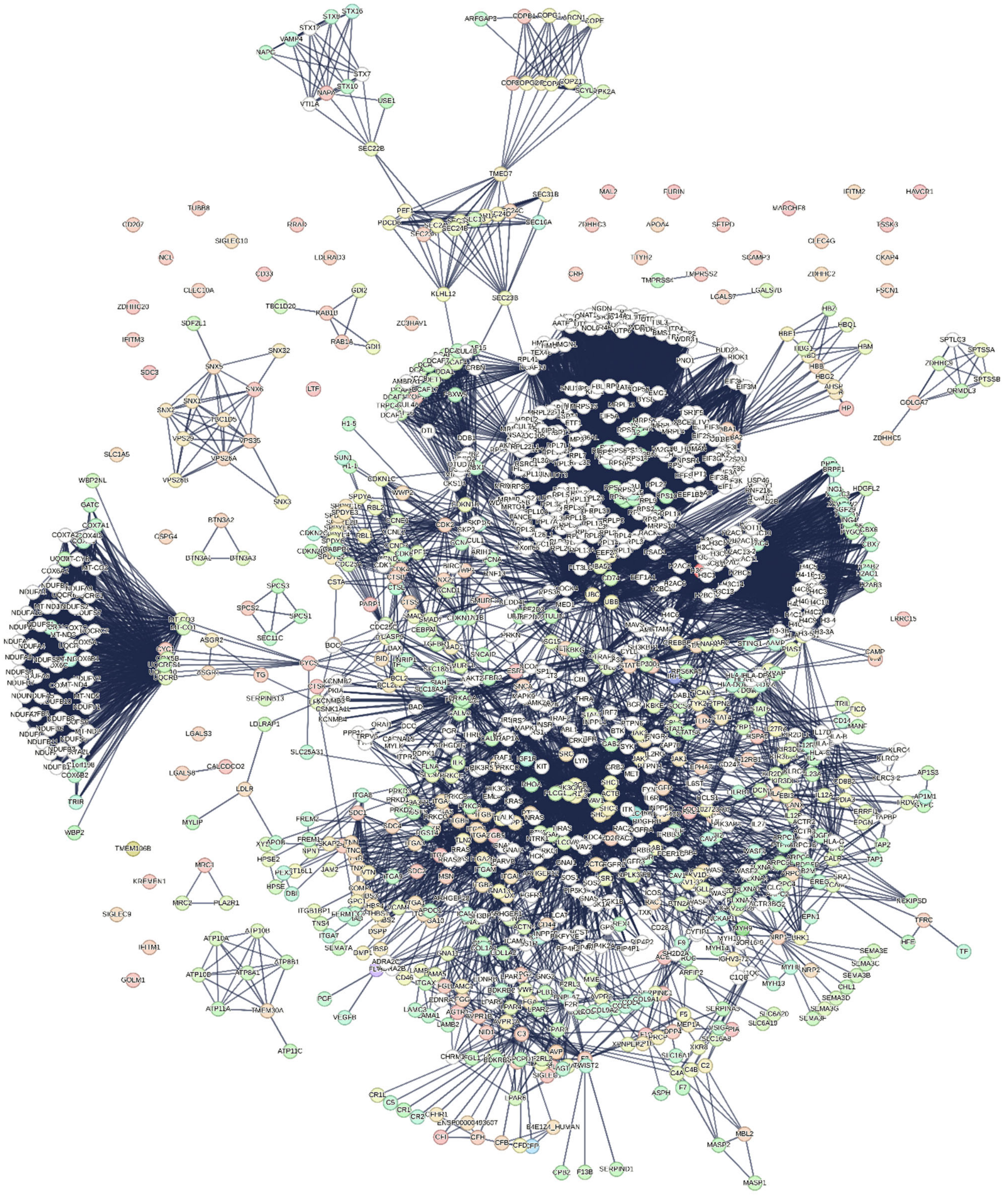

Figure S4. Functional enrichment of the interactome in the figure 2S. Confidence score: 0.900; 6 channels (No Textmining); 500 1st order proteins + 500 2nd order proteins. Topological parameters - number of nodes: 1146; number of edges: 17591; average node degree: 30.7; avg. local clustering coefficient: 0.667; expected number of edges: 9463; PPI enrichment *p*-value: $< 1.0 \times 10^{-16}$.

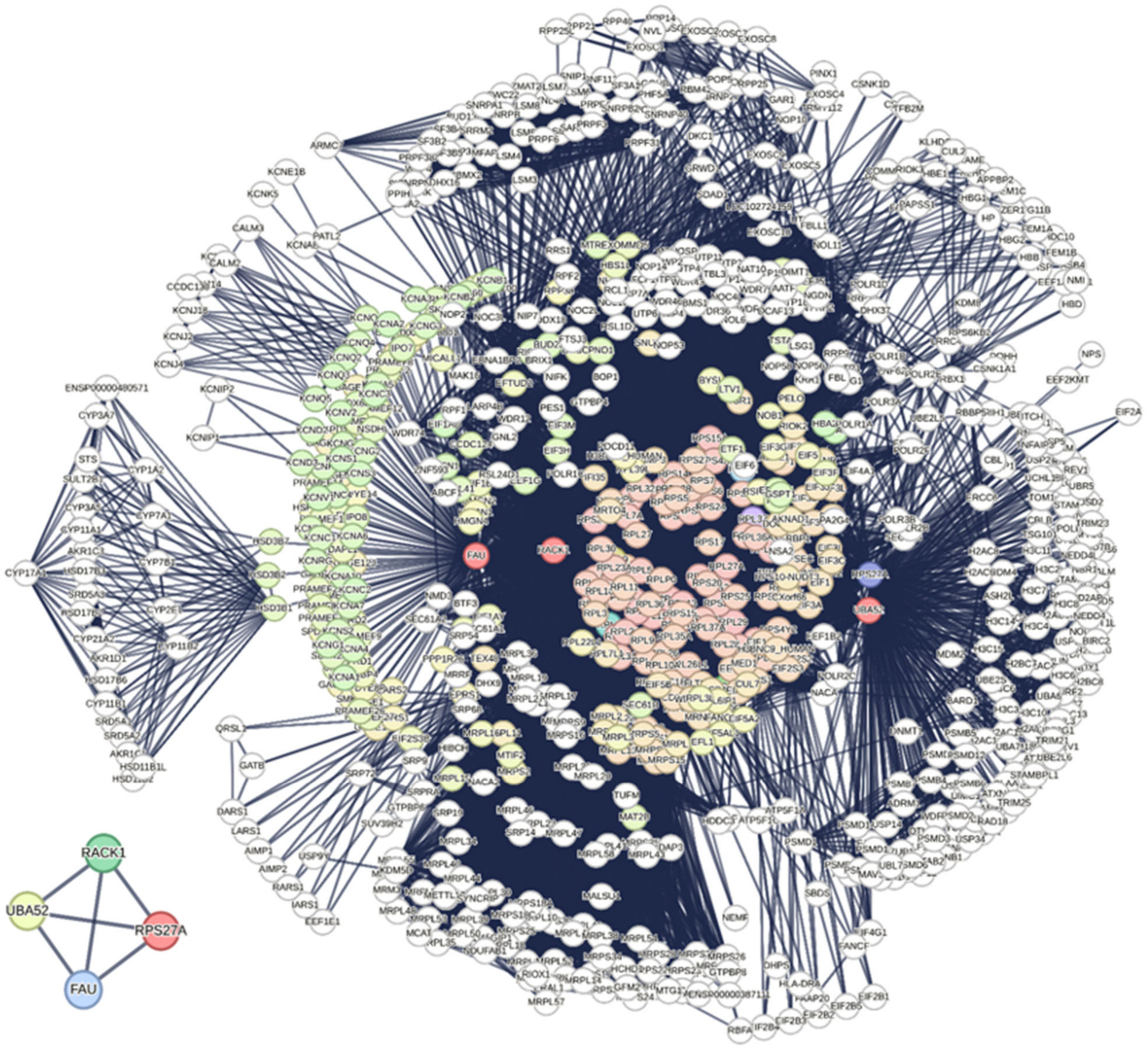

Figure S5. The world of FAU, RACK1, UBA52, and RPS27A (degree: 202, 147, 278, 297, respectively). The figure shows the control that the four proteins exert on 793 nodes of the interactome-1060. We got this network from the interactome-1060 using the STRING action, which allows us to focus on a node and re-center the network around it, showing all the functionally involved proteins. In red, FAU, RACK1, UB52 and in blue RPS27A. Number of nodes: 793; avg. local clustering coefficient: 0.81; PPI enrichment *p*-value: $< 1.0x10^{-16}$; confidence score: 0.900; 6 channels open (no Text mining data). The small graph at the bottom left shows the real connectivity relationships between the four nodes. Here, only the Experiments channel was open with a confidence score of 0.900 to evidence the robustness and physical existence of the connection system. We have placed a specific focus on the regulatory mechanisms employed by these proteins, while the ribosomal system plays a central role in the interactome. FAU controls the potassium voltage-gated channel system (in green), then through three enzymes of the 3 beta-hydroxysteroid dehydrogenases, controls the biosynthesis of steroid hormones (to the left, in white). UBA52 and RPS27A work together in controlling cellular proteins (to the right, in white) for degradation by the 26S proteasome, in the maintenance of chromatin structure, regulating gene expression through histones, and the stress response.

§ S1 -Meaning of the term one-to-one used in this study.

Structural biology and biochemistry are fields characterized by intricate dynamics, and many factors influence how proteins interact within a cellular environment. Protein interactions are not simple or linear but involve multiple factors, such as cellular context, post-translational modifications, and the dynamic nature of proteins. Context is essential, as proteins can behave differently based on their cellular environment and other molecules.

In this study, the term "one-to-one interaction" does not refer to interactions that can lead to the formation of protein complexes that perform specific tasks. It is used to describe the specific binding of the S1 subunit to unique target proteins, regardless of whether they belong to more complex systems. However, it is important to recognize the complexity inherent in protein interactions. The cellular environment and various proteoforms influences interactions resulting from post-translational modifications and alternative splicing. This means that the protein that operates in the cellular context is no longer the one encoded in the gene. These modifications change the protein's structure by chemically altering it, resulting in a change in conformation. Therefore, its function is also affected. The dynamic nature of proteins means they can exist in multiple conformations and oligomeric states, complicating our understanding of interactions. This dynamism of proteins complicates our ability to characterize interactions straightforwardly and highlights the need for sophisticated methodologies to study them.

Current literature lacks comprehensive in vivo data that detail the chronological order and the specific conditions under which these interactions occur. While in vitro studies provide foundational insights, those in vivo may not fully capture the complexities of protein behavior in a living system. As many interactions remain poorly characterized, further research employing advanced methodologies is needed to understand the 'where,' 'how,' and 'when' of protein interactions in a cellular context. Novel approaches would help bridge the gap between our current understanding and the complexities of biological interactions.

By acknowledging this complexity, we can provide a more nuanced understanding of our findings and the limitations of current knowledge in structural biology. The complexities of protein dynamics and interactions require careful consideration and further investigation, particularly when we seek to understand their roles within biological systems. A molecular approach to framing complexity, as in this study, is a necessary effort to understand what is happening during covid. We should know the limitations of current methodologies and data, such as reliance on in vitro studies that may not fully capture the complexity of in vivo interactions. Recognizing that interactions can change over time and under different physiological conditions, meaning that any interaction may not universally apply.

However, several of the 12 one-to-one interacting proteins in the liver take part in various biological processes that may involve the formation of oligomers or larger molecular complexes. Here are some examples:

1. ACE2 (Angiotensin-converting enzyme 2) - Involved in the formation of complexes, particularly as a receptor for the SARS-CoV-2 virus, which includes interactions with viral proteins.
2. DPP4 (Dipeptidyl Peptidase 4) - Can form complexes with various proteins and is involved in signaling pathways.
3. PRC1 (Protein Regulator of Cytokinesis 1) - Involved in the formation of protein complexes during cell division.
4. S100A8 - This protein forms hetero-oligomers with S100A9, contributing to the formation of the complex known as calprotectin.
5. TLN1 (Talin 1) - Functions in cell adhesion and forms complexes with integrins and other proteins.
6. TLR4 (Toll-like receptor 4) - Forms complexes with other proteins as part of the immune response, particularly in recognizing pathogens.

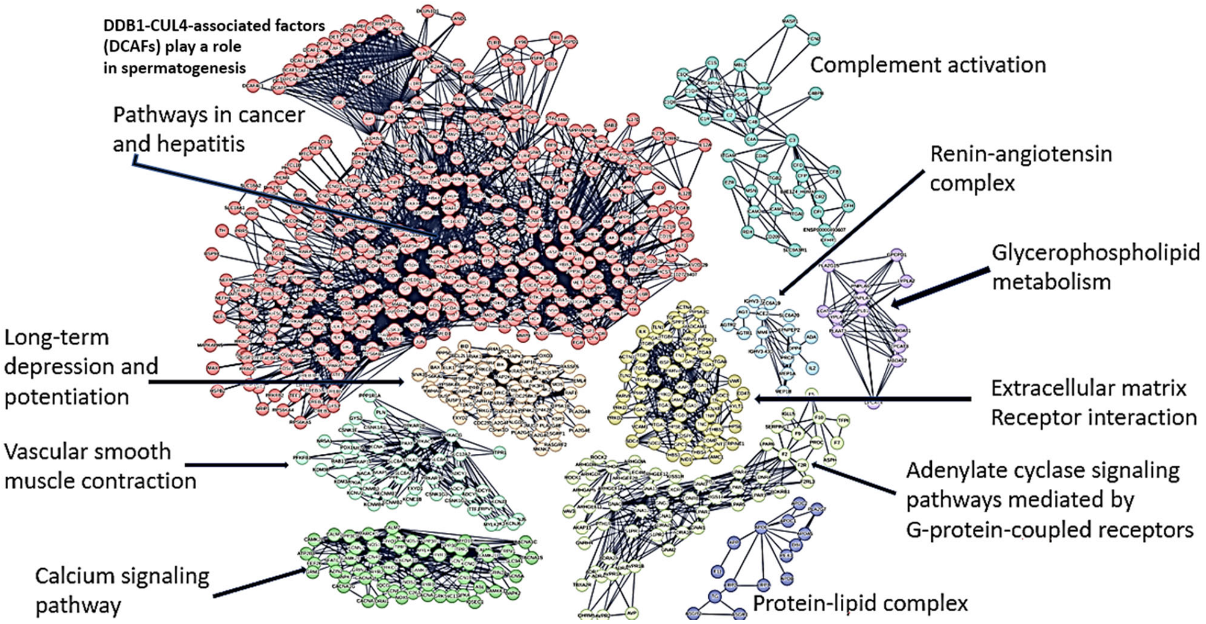

Figure S6. The most important functional subgraphs of the interactome-814. To visually highlight their quantitative impact, I removed all the existing connections between the subgraphs. The interactome core (in red) includes several important functions that are tightly connected to each other so that they can easily exert cross-functional influences.

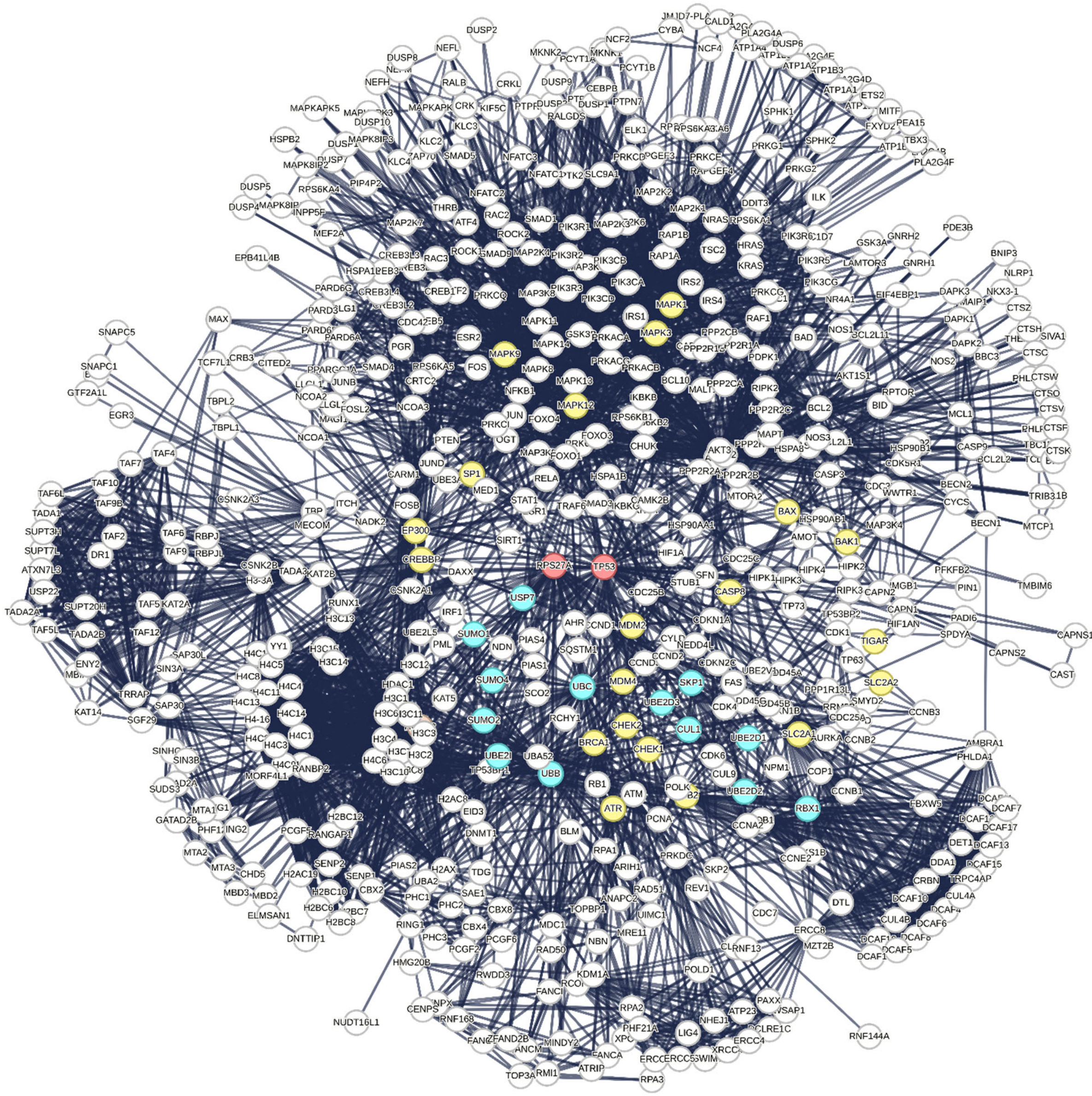

Figure S7. The world of TP53 and RPS27A. The figure shows the interactome of 588 proteins controlled by the two proteins. We got this network from the interactome-814 using the STRING action, which allowed us to focus and re-center the network around TP53, showing all the functionally involved proteins. In red TP53 and RPS27A. Both proteins have a tight connection. Number of nodes: 588; number of edges: 4804; avg. local clustering coefficient: 0.639; PPI enrichment p-value: $< 1.0x10^{-16}$; confidence score: 0.900; 6 channels open (no Text mining data). In yellow, the first level of proteins controlled by TP53, as mentioned in the article's text, and in light blue those controlled by RPS27A. Among the most interesting processes of this interactome we find: hsa05200 Pathways in cancer, 116 proteins, *p*-value: $5.64x^{-58}$; hsa05203 Viral carcinogenesis, 70 proteins, *p*-value: $3.81x^{-47}$; hsa04931 Insulin resistance, 46 proteins, *p*-value: $4.21x^{-33}$; hsa04152 AMPK signaling pathway, 45 proteins, *p*-value: $2.79x^{-30}$; hsa05225 Hepatocellular carcinoma, 43 proteins, *p*-value: $3.26x^{-24}$; hsa04611 Platelet activation, 37 proteins, *p*-value: $1.51x^{-22}$; HSA-2219528 PI3K/AKT, Signaling in Cancer, 33 proteins, *p*-value: $1.01x^{-19}$; DOID:0050686 Organ system cancer, 71 proteins, p-value: $3.23x^{-13}$; DOID:0080355 Hepatobiliary system cancer; 15 proteins, *p*-value:$6.42x^{-06}$. All strength values were between 0.8 and 1.3.
